# Marine heatwave conditions drive carryover effects in a temperate sponge microbiome and developmental performance

**DOI:** 10.1098/rspb.2022.2539

**Published:** 2023-06-14

**Authors:** Francesca Strano, Valerio Micaroni, Torsten Thomas, Lisa Woods, Simon K. Davy, James J. Bell

**Affiliations:** ^1^ School of Biological Sciences, Victoria University of Wellington, Wellington 6140, New Zealand; ^2^ School of Mathematics and Statistics, Victoria University of Wellington, Wellington 6140, New Zealand; ^3^ Centre for Marine Science and Innovation, University of New South Wales, Sydney 2052, Australia; ^4^ School of Biological, Earth and Environmental Sciences, University of New South Wales, Sydney 2052, Australia

**Keywords:** complex life cycle, metamorphosis, vertical transfer, microbiology, developmental plasticity, adaptation

## Abstract

Marine heatwaves are increasingly subjecting organisms to unprecedented stressful conditions, but the biological consequences of these events are still poorly understood. Here we experimentally tested the presence of carryover effects of heatwave conditions on the larval microbiome, settlers growth rate and metamorphosis duration of the temperate sponge *Crella incrustans*. The microbial community of adult sponges changed significantly after ten days at 21°C. There was a relative decrease in symbiotic bacteria, and an increase in stress-associated bacteria. Sponge larvae derived from control sponges were mainly characterised by a few bacterial taxa also abundant in adults, confirming the occurrence of vertical transmission. The microbial community of sponge larvae derived from heatwave-exposed sponges showed significant increase in the endosymbiotic bacteria *Rubritalea marina.* Settlers derived from heatwave-exposed sponges had a greater growth rate under prolonged heatwave conditions (20 days at 21°C) compared to settlers derived from control sponges exposed to the same conditions. Moreover, settler metamorphosis was significantly delayed at 21°C. These results show, for the first time, the occurrence of heatwave-induced carryover effects across life-stages in sponges and highlight the potential role of selective vertical transmission of microbes in sponge resilience to extreme thermal events.

## 1. Background

Marine heat waves (MHWs) are periods of extreme sea surface temperatures that can occur at a variety of scales, from a few miles to an entire ocean, and can last from a few days to years [1]. Although our knowledge of the physical characteristics of MHWs has rapidly increased over the past decade, the effects of MHWs on biological processes are still not well understood and require particular attention [2]. MHWs have already caused massive coral bleaching events in the tropics [3] and mass mortalities of habitat-forming invertebrates, including sponges and gorgonians, in temperate and polar seas [4–6], and the frequency and intensity of MHWs are predicted to increase [7].

The advantages for sessile benthic organisms of having a planktonic larval stage include high dispersal potential and increased ability to withstand local extinction [8,9]. Pre- and post-settlement larval phases are critical stages to consider when trying to understand the effects of climate change on marine invertebrates [10]. Carryover effects exist when an individual's previous experience explains its current performance, and this can occur between developmental and life-history stages [11]. Temperature is regarded as an important environmental cue for developmental processes [12] and extreme temperatures can have substantial effects on an organism's metamorphosis, and influence timing, duration, developmental changes and energy costs, with consequences for the fitness of metamorphosing individuals [13].

Sponges are some of the most abundant organisms in benthic marine environments [14], and are present at all latitudes, from coastal waters to the deep sea, with more than 9200 described species [15]. Several studies have correlated the phenology of coastal sponge species to sea surface temperature. In particular, annual temperature peaks have been correlated with the highest percentage of reproductive individuals, as well as to greater oocyte size and density, and the occurrence of larval blooms of several sponges in temperate seas [16–20]. Therefore, it is likely that extreme thermal events will affect the developmental processes along with pre- and post-settlement stages of temperate sponges.

Sponges are characterized by complex microbial symbiotic communities that are species-specific and tend to be stable in the face of environmental change [21–23]. The combination of the sponge host and microbial symbionts is referred to as the ‘holobiont’ [24], which in some cases, has been identified to be the result of co-evolutionary processes [25]. Sponge symbiotic microbial communities can contribute to sponge metabolism and the production of secondary metabolites [26]. These compounds can deter predators and give sponges advantages in spatial competition [27].

Vertical transmission occurs when microbes belonging to a parental sponge are transferred to gametes or larvae [28–31] and it is regarded as a fundamental process in ensuring the persistence of some components of the holobiont across generations [32]. MHWs have been shown to cause microbial dysbiosis and tissue necrosis in several temperate sponges [33–36]. However, whether thermally driven microbial shifts affect vertical transmission and the larval microbial community in sponges is currently unknown.

*Crella incrustans* is an abundant subtidal sponge distributed in temperate Pacific waters and is increasingly considered a model organism in eco-physiological studies [37–39]. Like many Demospongiae in temperate seas [40], *C. incrustans* is a simultaneous hermaphrodite that broods and releases larvae during the summer months [41]. In this study, we experimentally exposed *C. incrustans* to temperatures recorded during a recent MHW in New Zealand, which occurred during the summer of 2018 and lasted for 11 days at 20.5 ± 0.64°C (daily mean and s.d., in Wellington Harbour). The specific aims of our study were: (1) to identify any modifications in morphology and microbial community of adult sponges under heatwave conditions; (2) to assess carryover effects of parental changes on the larval microbial community, and (3) on survival, growth and metamorphosis rates of early settlers grown under prolonged heatwave temperatures.

## 2. Methods

**Figure 1.**
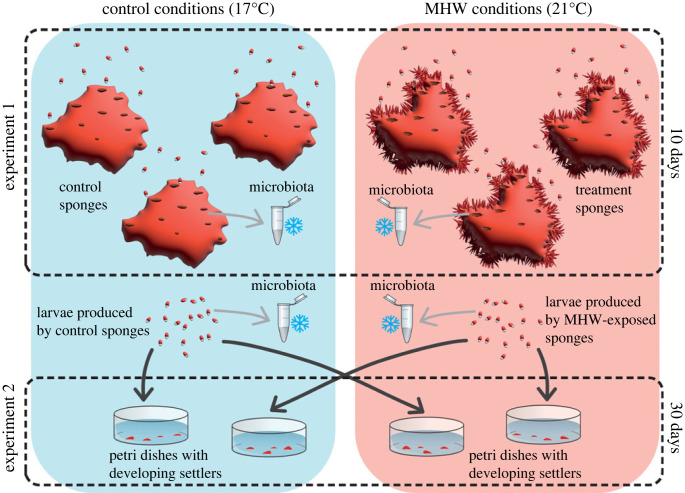
Schematic representation of the experiments and sampling design. Sponge larvae were randomly sampled from adult sponges in control or MHW conditions, either preserved for microbial community analyses or exposed to further control or prolonged MHW conditions.

To test our hypothesis, we performed two experiments (figure 1). In experiment 1, we exposed reproductive individuals of *C. incrustans* to heatwave conditions (10 days at 21°C) and assessed changes in sponge morphology and in the associated microbial community of adults and larvae. In experiment 2, we sampled sponge larvae generated during the first experiment, exposed them to prolonged heatwave conditions (up to 30 days at 21°C) and assessed the mortality, growth rate and duration of metamorphosis of the resulting settlers.

### (a) Experiment 1: effects of heatwave conditions on adult sponges

On the last week of September 2020, samples of *C. incrustans* (approx. 25 cm^2^) were haphazardly collected from separate sponges, at least 5 m apart, by SCUBA diving at 3 m in Wellington Harbour, New Zealand (41°17′32.1″S, 174°50′00.7″E). Sponges were collected under Special Permit (711-8730069, Ministry of Primary Industry, NZ). To allow sponge tissue regeneration, sponges were tied with elastic wire to ceramic tiles and left underwater at the same sampling site and depth for three months after collection. On the first week of January 2021, sponges (*n* = 48) were moved to the Wellington University Coastal Ecology Laboratory and left to acclimate in experimental tanks (*n* = 8 sponges per tank) for eight days (acclimation period).

Sponges were kept in 6 l flow-through tanks (*n* = 3 control and *n* = 3 heatwave conditions), with a flow rate of approximately 30 ± 2.5 l h^−1^ of 10 µm filtered seawater (similar to [42]). Aquarium heaters, (200 W, Eheim, Germany) and a chiller (HC-500A, Hailea, China), interconnected with an aquarium temperature controller system (Apex Classic, Neptune Systems, USA) and a temperature probe (YSI Pro30, USA), were used to monitor and modify water temperature during the experiment (see electronic supplementary material, table S1 for experimental temperatures and electronic supplementary material, figure S1 for experimental set-up).

During the acclimation period, the water temperature was maintained at a similar temperature as the sampling site on the day of collection (16.8 ± 0.2°C, mean and s.d. at the sampling site and 17.3 ± 0.1°C mean and s.d. under laboratory conditions; electronic supplementary material, table S1). After the acclimation period, the water temperature was increased 0.5°C each day [43,44] for 10 days, up to 21°C, in treatment tanks. Sponges were then maintained at 21°C for 10 days. These conditions are analogous to the temperatures recorded in Wellington Harbour during the MHW that occurred during January 2018 (electronic supplementary material, figure S2).

To facilitate the collection of sponge larvae from experimental tanks, each sponge was placed within a larval trap with 48 µm mesh (electronic supplementary material, figure S1). Larval release inside larval traps was monitored daily. The occurrence of sponge morphological modification in response to high temperatures were recorded every day. Sponges with ≥25% necrotic tissue were considered dead and were removed from the experimental tanks [39].

### (b) Microbial community analysis of adult and larval sponges

Samples of adult sponges and sponge larvae were collected for microbial community characterization after 10 days under control and treatment conditions (experiment 1, figure 1). Samples of adult sponges (3 cm^3^, including pinacoderm, mesohyl and choanoderm) were sampled with a sterile scalpel and forceps from random sponges in control (*n* = 5) and MHW conditions (*n* = 10), including sponges presenting tissue regression and necrosis (*n* = 5 and *n* = 5, respectively). Samples were then flash-frozen in liquid nitrogen and stored at −80°C.

Sponge larvae generated from different sponges either in control or heatwave conditions were collected by sterile Pasteur pipettes, transferred into sterile tubes, washed three times in filtered seawater (0.2 µm), snap-frozen in liquid nitrogen and stored at −80°C.

Microbial DNA was extracted from adult sponge samples (*n* = 15) and groups of 20 larvae (*n* = 8 groups of larvae that were derived from multiple control adult sponges and *n* = 8 groups of larvae derived from multiple MHW-exposed adult sponges) using a DNeasy PowerSoil Pro Kit following the manufacturer's protocol (Qiagen, Germany). We used Nanodrop and gel electrophoresis on 1% agarose gels containing 0.5 µg ml^−1^ SYBR Safe Gel Stain (Invitrogen, Carlsbad, USA) to assess the concentration and quality of the extracted gDNA.

Amplicon library construction and sequencing were performed by Macrogen (Seoul, South Korea) on the MiSeq platform (Illumina). To target the V3–V4 region of the 16S rRNA gene, we used the primers Bakt_341F (5′-CCTACGGGNGGCWGCAG-3′) and Bakt_805R (5′-GACTACHVGGGTATCTAATCC-3′) [45]. For library preparation, gDNA was PCR amplified with reaction buffer, 1 mM of dNTP mix, 500 nM each primer, and Herculase II Fusion DNA polymerase (Agilent Technologies, Santa Clara, CA). PCR conditions were: 3 min at 95°C for initial denaturation, followed by 25 cycles of denaturation at 95°C for 30 s, annealing at 55°C for 30 s and elongation at 72°C for 30 s, followed by a final extension (5 min at 72°C). Resulting PCR products were purified with AMPure XP beads (Agencourt Bioscience, Beverly, MA) and quantified using qPCR according to the qPCR quantification protocol guide (KAPA Library Quantification Kit Illumina Platforms). Purified amplicons were indexed using the Nextera XT DNA Library Preparation Kit (Illumina). The final purified product was quantified using the TapeStation D1000 ScreenTape (Agilent Technologies, Waldbronn, Germany).

Raw sequences were pre-processed and zero-distance operational taxonomic units (zOTUs) were classified [46]. Raw sequences were filtered, trimmed and primers were removed using USEARCH v. 11.0.667 and TRIMMOMATIC version 0.38. Unique sequences were generated using *unoise3* and chimera were removed with *uchime2*. For taxonomic assignment of zOTUs, the Bayesian Last Common Ancestor (BLCA) algorithm was applied to compare sequences against the Genome Taxonomy Database, release 95 [46,47].

### (c) Experiment 2: effects of prolonged heatwave conditions on sponge settlers

Concurrently with the experiment on adult individuals (experiment 1; figure 1), a heatwave-exposure experiment was performed on the post-settlement developmental stages of *C. incrustans* (experiment 2; figure 1), also defined as ‘sponge settlers’ [48]. To assess carryover effects of MHW conditions on survival, growth rate and duration of metamorphosis of sponge settlers under prolonged heatwave conditions, newly released larvae from experiment 1 were collected from random adults separately for each treatment with sterile Pasteur pipettes. After collection, each group of larvae (*n* = 10) was placed in a sterile Petri dish (34 mm in diameter), filled with 14 ml of FSW (0.2 µm) and placed in control or heatwave conditions, in water baths within random experimental tanks (electronic supplementary material, figure S1). Larvae generated by sponges under control or heatwave conditions were, in turn, exposed to control or high temperatures (i.e. 21°C), resulting in four combinations of treatments.

The overall number of Petri dishes and settlers generated was *n* = 32 and *n* = 165, respectively. Settler health and the formation of the first osculum, which was considered the completion of metamorphosis [49], were monitored under a dissecting microscope (Olympus SZ61, 45×) every day, up to 30 days after settlement. Filtered (0.2 µm) and pre-acclimatized (17°C or 21°C) seawater was replaced in each Petri dish during daily monitoring of settlers. To calculate the growth rate of settlers under the four treatment combinations (i.e. larvae derived from control and heatwave-exposed sponges and placed in heatwave or control conditions), five random settlers, within three random Petri dishes, were selected on the day of settlement (*n* = 60 settlers and *n* = 12 Petri dishes) and photographed with a Canon EOS 70D digital camera under a dissecting microscope (Olympus SZ61) every two days for 20 days. To calculate the surface area of sponge settlers, pictures were analysed with ImageJ (v. 1.51j8, Rasband, National Institute of Health).

### (d) Statistical analyses

All statistical analyses were carried out in R 4.1.0 [50]. Kaplan–Meier analyses were performed to calculate the probability of the occurrence of tissue regression or necrosis, and sponge and settler probability of survival under control and heatwave conditions. The relative *p*-values were obtained with *survfit* of the package *survival* [51].

A mixed effects logistic regression (R package *lme4*) was used to calculate differences in sponges producing and not producing larvae between the two temperature treatments [52]. The dependent variable was the presence (or absence) of larvae, fixed factors used in the model were ‘parental sponge condition’ (control or heatwave temperatures), whereas ‘experimental tank’ was a random factor. Effects of fixed factors were tested by ANOVA with the package *car* [53].

Differences between sponge and larval microbial communities under different thermal treatments (control or heatwave temperatures) were explored using non-metric multidimensional scaling (nMDS) based on Bray–Curtis dissimilarity and by principal coordinates analysis based on the Sørensen dissimilarity index. To calculate significant differences in sponge and larval microbial communities under control or heatwave temperatures, we used generalized linear models for multivariate abundance data (*manyglm*) implemented in the R package *mvabund* [54]. To test the model, *anova* was used, with 999 bootstrap interactions and the ‘montecarlo’ method of resampling [54]. To identify significant changes in the composition of microbiomes, the *ancombc* package was used and *p*-values were Benjamini-Hochberg corrected [55].

Significant changes in settler area were estimated by linear mixed models with the *lme4* package [52]. To meet the normality assumptions, the area values were square-root transformed. The fixed factors used in the model were ‘time’, ‘parental sponge condition’ (control or heatwave temperatures) and ‘settler condition’ (control or heatwave temperatures), whereas ‘Petri dish’ and ‘settler’ were random factors. Effects of fixed and random factors were tested by *anova* and *ranova,* with the package *lmerTest* [56]. Pairwise comparisons were calculated on estimated marginal means using the *emmeans* and *emtrends* functions of the *emmeans* package [57], and *p*-values were corrected with the Benjamini–Hochberg procedure.

Significant differences in the duration of metamorphosis for settlers exposed to control and heatwave conditions were calculated by linear mixed models with the *lme4* package [52]. The fixed factors in the model were the ‘mean number of days until the formation of an osculum in each Petri dish’, ‘parental conditions’ and ‘settler conditions’, while ‘tank’ was a random factor. Effects of fixed and random factors were tested by ANOVA and RANOVA [56].

## 3. Results

### (a) Effects of heatwave conditions on adult sponge morphology and survival

All adult sponges showed tissue regression and 37.5% necrosis in response to MHW conditions. Kaplan–Meier analyses showed a significant decrease in the probability of morphological integrity (absence of tissue regression; *p* < 0.0001) and sponge survival (absence of necrosis; *p* = 0.001) after 10 days at 21°C (electronic supplementary material, figure S3 and table S2).

Sponges produced larvae in both treatments; 25% of sponges produced larvae in the control and 45.8% in heatwave conditions, respectively, although this difference was not significant (*p* = 0.14) (electronic supplementary material, table S3).

### (b) Effects of heatwave conditions on adult and larval sponge microbiomes

**Figure 2.**
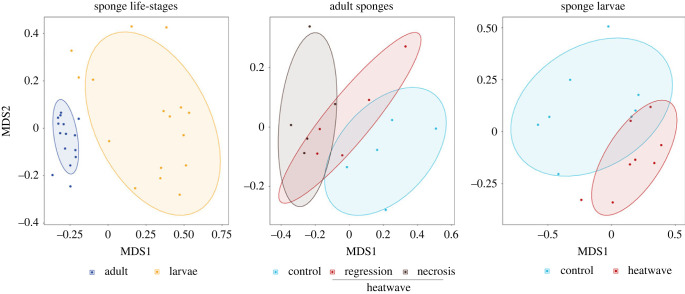
Non-metric multidimensional scaling (nMDS) based on Bray–Curtis dissimilarity representing: overall sponge and larval microbial communities; microbial communities of sponges in control and heatwave conditions (including sponges that presented tissue regression and necrosis); microbial communities of larvae produced by sponges in control and heatwave conditions.

A total of 5 679 582 16S rRNA gene raw reads, 1 899 871 clean reads and 1748 zOTUs (with 61 286.16 ± 13 485.01 as mean and s.d. of reads per sample) were obtained from adult and larval samples (electronic supplementary material, figure S4). Non-metric multidimensional scaling (nMDS) based on Bray–Curtis dissimilarity (figure 2) and generalized linear models for multivariate abundance data testing showed significant differences between sponge and larval microbial communities (*p* = 0.001; electronic supplementary material, table S4a).

Overall, the multivariate test showed that the microbial community of adult sponges in control conditions was significantly different from the microbial community of heatwave-exposed sponges (*p* = 0.001). Pairwise tests revealed significant differences in the microbial communities between sponges in control conditions and those showing tissue regression (*p* = 0.001) and necrotic tissue (*p* = 0.001) under heatwave conditions (electronic supplementary material, table S4b).

Principal component analysis based on the Sørensen dissimilarity index showed that these differences were not only driven by the relative abundances of zOTUs but also by changes in microbial community composition (electronic supplementary material, figure S5). The microbial community of sponge larvae showed higher beta-diversity than the adult microbial community (electronic supplementary material, figure S5).

**Figure 3.**
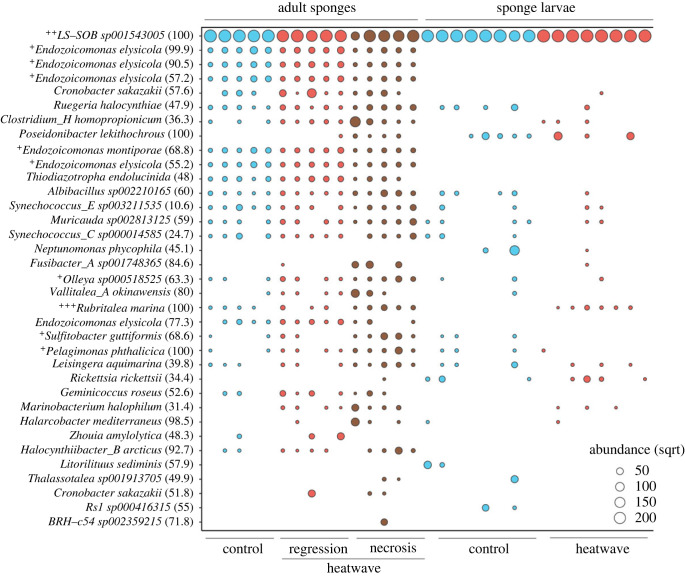
Bubble plot representing the relative abundance of zOTUs in control and heatwave conditions and across adult and larval sponge life-stages. The size of each bubble corresponds to the abundance of zOTUs standardized for the mean sequencing depth and square-root transformed. Numbers in brackets represent level of identity of zOTUs of each bacterial species. ANCOM-BC results at the species level (electronic supplementary material, table S9) are also represented: (^+^) indicates significant differences between control sponges and sponges exhibiting necrosis; (^++^) indicates significant differences between control sponges and sponges exhibiting tissue regression; (^+++^) indicates significant differences between larvae produced by control sponges and sponges exhibiting tissue regression. Only zOTUs more abundant than 0.001 are represented.

The microbial community of *C. incrustans* in control conditions was mainly composed of *Gammaproteobacteria* belonging to the *Areniclellales* order [58], previously called *UBA10353*, and *LS-SOB sp001543005* species, representing 76.4–94.8% of the overall relative read abundance of the community (figure 3). Next most abundant was *Pseudomonadales* and *Enterobacterales*, which represented up to 19.8% and 1.4% of the microbial community at the order level, respectively.

Compared to sponges in control conditions, heatwave-exposed sponges with tissue regression were characterized by a significant decrease in *Arenicellales* (*p* = 0.01; electronic supplementary material, table S5). There was also a significant increase of *Flavobacteriales* (*p* = 0.01), representing 0.8% in control sponges and up to 7.3% of the microbial community in sponges with temperature-induced tissue regression.

Sponges that showed tissue necrosis in response to MHW conditions were characterized by a significant decrease in *Arenicellales* (*p* < 0.001), which represented 12.4–74.1% of the microbial community. There was also a significant increase in *Micavibrionales* (*p* < 0.001; electronic supplementary material, table S5). In some cases, *Clostridiales* represented up to 41% of the microbial community.

We found significant differences between the microbial communities of larvae produced by control and heatwave-exposed sponges (*p* = 0.001; figure 1; electronic supplementary material, table S4c). The microbiome of larvae produced by control sponges was comprised 49.8–99.1% *Arenicellales* (*LS-SOB sp001543005* species; figure 3), plus *Pseudomonadales* and *Enterobacterales*, which comprised up to 32% and 8.9% of the microbial community, respectively. The microbial community of larvae produced by heatwave-exposed sponges that displayed tissue regression had a higher relative abundance of *Arenicellales* (86–98.9%). Additionally, the microbial community of larvae produced by heatwave-exposed sponges was characterized by a significant increase in the bacterium *Rubritalea marina* (*p* = 0.01), which belongs to the class *Verrucomicrobiae* (figure 3; electronic supplementary material, table S5).

### (c) Effects of prolonged heatwave conditions on sponge settlers

Of a total 165 sponge settlers across treatment and control conditions, only 14 died within 30 days after settlement. Overall, when considering settlers derived from control and heatwave-exposed sponges, 4% of settlers died in control and 12% under high-temperature conditions. A cut off value of *p* ≤ 0.05 was interpreted as significant and according to the Kaplan–Meier analysis, MHW conditions significantly decreased the probability of survival of sponge settlers (*p* = 0.043) (electronic supplementary material, figure S6 and table S6*a*). There was no statistical support for a significant effect of parental treatment on settler mortality (*p* = 0.3) (electronic supplementary material, table S6*b*).

**Figure 4.**
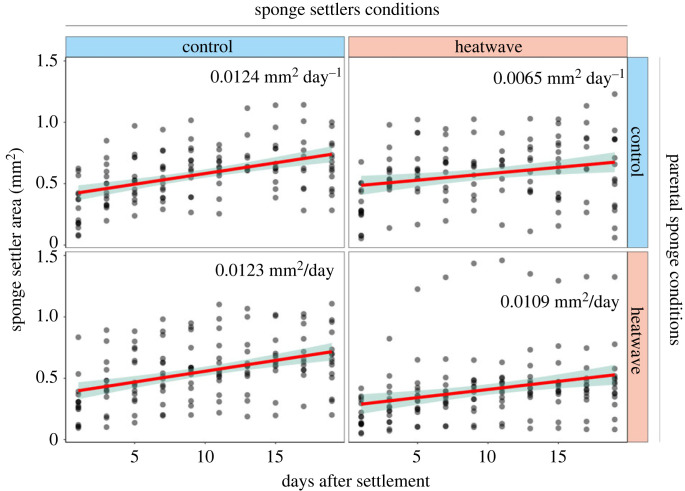
Scatter plots and relative linear regressions representing the growth rate of sponge settlers originating from control or heatwave-exposed sponges and in control or prolonged heatwave conditions, for up to 20 days of experimental treatment.

Settler area increased significantly over time under all treatments (*F*_1, 487.1_ = 241; *p* < 0.0001; figure 4; electronic supplementary material, table S7). Settler growth rate was significantly influenced by treatment (*F*_1, 487.1_ = 7.2; *p* = 0.007). MHW conditions experienced by the parental sponge also influenced settler growth rate (*F*_1, 9.9_ = 4.9; *p* = 0.051). In particular, settlers derived from control sponges and exposed to control conditions grew significantly faster than settlers exposed to heatwave temperatures (*p* = 0.005); their growth rates were 0.0124 mm^2^ day^−1^ and 0.0065 mm^2^ day^−1^, respectively. By contrast, settlers derived from heatwave-exposed sponges showed no difference in growth rate (*p* = 0.446) when exposed to control (0.0123 mm^2^ day^−1^) or heatwave conditions (0.0109 mm^2^ day^−1^). Settlers derived from MHW-exposed or control sponges showed significantly different growth rates (*p* = 0.048) when exposed to treatment temperatures but not control conditions (*p* = 0.964).

**Figure 5.**
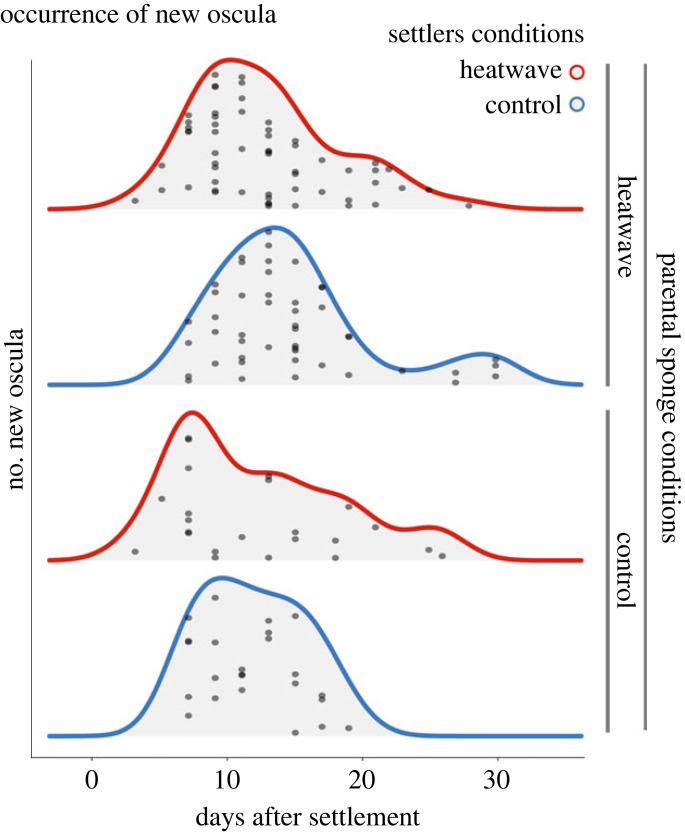
Ridgeline plots showing the occurrence of new oscula in sponge settlers for up to 30 days of experimental treatment.

The duration of settlers metamorphosis (figure 5) under prolonged heatwave conditions was significantly (*p* = 0.046) influenced by parental conditions (electronic supplementary material, table S8).

## 4. Discussion

Extreme temperatures occurring during MHWs can affect biological, physiological, and behavioural processes, but for most organisms, these aspects have not yet been studied [2]. Our study showed significant impacts of heatwave conditions not only on sponge morphology and microbial community, but also our results revealed the occurrence of carryover effects on sponge early stages at the microbial and developmental levels.

### (a) Heatwave conditions significantly reduce tissue integrity and survival probability in adult sponges

Heatwave conditions significantly affected the morphology and survival of *C. incrustans*. All sponges exposed to 21°C for 10 days showed tissue regression in response to thermal stress and 37.5% of them also suffered necrosis. Sponges can display high levels of phenotypic plasticity in response to environmental conditions [59,60]. In some cases, sponges can undergo specific morphological modifications to overcome periods of physiological stress [61]. Tissue regression is known as a reversible morphological modification that some sponge species display in response to thermal stress [39,62,63]. Tissue regression involves an internal and external reorganization of sponge anatomy, including the reabsorption of the aquiferous system and, in some cases, reproductive structures including embryos and larvae [60]. *Crella incrustans* is a viviparous sponge that broods embryos and releases fully developed larvae through the canals of the aquiferous system during the Australasian summer [41]. Heatwave conditions and tissue regression did not inhibit *C. incrustans* from releasing larvae and we detected no significant effects of these conditions on larval release. Previous histological observations of *C. incrustans* found that only 57% of sponges sampled between January and March 2020 (*n* = 30) had embryos at different developmental stages [41]. This indicates that not all of the sponges we collected released larvae during experiment 1, and this probably reflects natural variability present in the source population. In some cases, spawning events under stressful conditions are thought to be the last chance that organisms may have to escape an environmental stressor [64]. Larval release under heatwave-induced tissue regression may represent a reproductive strategy to overcome MHWs in *C. incrustans*.

The timing of MHWs in relation to organism phenology is an important aspect that needs further investigation. Between 2015 and 2016 a winter marine heatwave was correlated with failed spawning in several marine invertebrates in the Northeastern Pacific [65]. Considering that tissue regression is known as a morphological response of sponges to extreme temperatures in both experimental and field conditions [62,66], marine heatwaves occurring at the beginning of the reproductive period have the potential to adversely impact early gametogenesis and embryogenesis in sponges by causing the reabsorption of reproductive structures and consequently may disrupt the entire phenological event. It will be important in the future to examine the effects of the MHW timing on phenological events such as gametogenesis and embryogenesis not only in sponges but also in other marine organisms.

### (b) Heatwave conditions modify sponge and larval microbiome

Along with necrosis, tissue regression and mortality, heatwave conditions caused significant changes in the microbiome of adult sponges, with carryover effects on the larval microbial community (figures 2 and 3).

*C. incrustans* can be considered a ‘low microbial abundance’ sponge [67], since its microbiome is characterized by a relatively low microbial diversity, dominated by *Gammaproteobacteria* belonging the *LS-SOB* family (order *Arenicellales*). Bacterial taxa belonging to the *Arenicellales* were previously found also to be associated with some deep-sea [68–70] and coastal sponges [71,72]. Metagenomic sequencing of coastal and deep-sea sponges revealed that sulfur oxidation by *Arenicellales* is potentially relevant to sponge metabolism [68,73]. In the sponge *Mycale hentscheli*, *Arenicellales* are known to produce defensive polyketide compounds [72]. Considering the high metabolic potential of *Arenicellales* associated with other sponge species [72,73], the high abundance of *LS-SOB sp001543005* (figure 3) in both adults and larvae suggests that this microbe plays an important role in the biology of *C. incrustans*.

In adult *C. incrustans*, heatwave conditions led to a relative increase in *Flavobacteriales*. Bacteria belonging to this order are known to be opportunistic and primarily non-symbiotic bacteria, that increase in abundance during physiological stress and disease in sponges [74,75], as well as other marine organisms such as coralline algae and corals [76–78].

In addition to high relative abundance of *Flavobacteriales*, sponges with tissue necrosis exhibited a relative increase in *Micavibrionales*, while in some cases, *Clostridiales* represented up to the 41% of the microbial community. Bacteria belonging to these orders are often found in the presence of temperature-driven necrosis in tropical and temperate sponges [34,79,80]. These groups are often characterized by motility and anaerobic metabolism [79,80]. In *C. incrustans*, the increased abundance of bacteria that are likely non-symbiotic and opportunistic was consistent with the occurrence of tissue decay and sponge mortality in response to heatwave conditions. Concurrently with the introduction of these opportunistic bacteria, adult sponges showed a relative decrease in symbiotic species including *LS-SOB sp001543005*. However, it should be noted that the relative decrease in symbiotic microorganisms may not be due to an absolute decrease in their population, but rather a result of the proliferation of opportunistic bacteria. In addition, microbial communities of thermally stressed sponges were highly divergent and dispersed than microbial communities of control sponges (electronic supplementary material, figure S5). These results are consistent with the ‘Anna Karenina principle’ for organisms-associated microbiomes, in which individuals displaying dysbiosis have higher variability in their microbial community composition compared to healthy individuals [81].

Heatwave-induced microbial modifications of adult sponges led to carryover effects on the larval microbial community. Our results provide support for the occurrence of vertical microbial transfer in *C. incrustans* and suggest that larval microbial communities exhibit greater variability than those of adults. Previous studies have also reported that in addition to bacteria dominating the adult microbiome, sponge larvae can present less abundant, stochastically transmitted bacteria [32]. This level of stochasticity in vertical transmission could account for the higher beta-diversity observed in the microbial community of *C. incrustans* larvae, and may provide high functional plasticity in response to various environmental conditions for the recruits. Furthermore, our results showed the occurrence of selective vertical transmission in sponge larvae released under heatwave conditions. Larvae produced by control sponges were characterized by a higher relative abundance of *Arenicellales*, followed by *Pseudomonadales* and *Enterobacterales*, as seen in adult sponges. By contrast, the microbial community of larvae released during the simulated MHW did not change in the same way as the microbiome of parental sponges. Indeed, sponge larvae produced under heatwave conditions showed a relative increase in *LS-SOB sp001543005,* that in thermally stressed adult sponges decreased substantially. These larvae were also characterized by a lower relative abundance of *Flavobacteriales*, that in contrast increased significantly in adult sponges exposed to high temperatures. In addition, the relative abundance of the bacterial species *Rubritalea marina* significantly increased in larvae produced under heatwave conditions (figure 3), despite not being an abundant species in parental sponges.

The microbial community of developing sponges is composed of both bacteria acquired from the microbial pool of the parental sponge and other bacteria acquired horizontally from the surrounding environment [31,32]. Generally, larval microbial community is compositionally similar to that of the parental sponge and the acquisition of symbionts from the environment starts after the opening of the first osculum in sponge settlers [32,82]. In *C. incrustans*, several bacterial taxa, such as bacteria belonging to the genus Endozoicomonas [83], seemed to be horizontally acquired during post-settlement stages. A recent study suggested that changes in pH and temperature may cause variations in the transmission of low-abundance microbial species and that these variations might be involved in sponge acclimatization to new environments [75]. *Rubritalea marina* belongs to the phylum *Verrucomicrobia* and was originally isolated from the Mediterranean sponge *Axinella polypoides* [84]. Bacteria belonging to the genus *Rubritalea* are often found in association with sponges, with several strains producing carotenoid pigments and squalene [85]. Considering the very low relative abundance of *R. marina* in adult *C. incrustans* and the consistent presence of this microbe in larvae derived from heatwave-exposed sponges, we suggest the occurrence of selective vertical transmission of this bacterium under high temperature conditions. The possible functions of *R. marina* during thermal stress in adult and the early stages of development in *C. incrustans* require further investigation.

### (c) Heatwave conditions have carryover effects on sponge early life-history traits

Our results showed that, despite prolonged heatwave conditions negatively affecting the survival of sponge settlers, the ones produced by heatwave-exposed sponges showed significant carryover effects on settler growth rate and metamorphosis duration.

Consistent with these results, previous experiments on *C. incrustans* showed an overall decrease in the survivability of sponge settlers under MHW conditions [39]. In addition, the growth rate of settlers produced by heatwave-exposed sponges was similar under high-temperature and control conditions. By contrast, the growth rate of settlers originating from control sponges was significantly impacted by heatwave temperatures (figure 4). It is important to note that certain outliers in settler growth rates, particularly under high temperatures, can arise from natural variability within the population in response to thermal stress. Future studies could shed light on possible differences in developmental performance of larvae derived from different parental sponges under high temperatures.

In several marine invertebrates, larval developmental history has been shown to influence the performance of early settlers [86]. For example, past studies showed that high temperature conditions during development and larval stages can have negative latent effects on post-settlement processes (e.g. growth and survival) in some echinoderm and mollusc species. In some cases, high temperatures have also caused positive carryover effects from one life-stage to the other, such as higher thermal resistance in amphipods [87], larger juveniles in the shortspined sea urchin [44] and higher survival rates in the Olympia oyster [88]. In *C. incrustans*, most settlers produced by heatwave-exposed sponges survived under heatwave temperatures and grew at similar rates to settlers maintained under control conditions. Considering that the experimental period of exposure for settlers (up to 30 days) was a much longer period compared to MHWs recorded in *C. incrustans*' natural habitat (5–12 days in the last 13 years in Wellington Harbour; electronic supplementary material, figure S2), we conclude that developmental exposure to heatwave conditions might have positive carryover effects on settler growth rate under heatwave conditions in this sponge species.

Parental exposure to MHW conditions caused carryover effects on the duration of metamorphosis of sponge settlers under prolonged heatwave conditions. Indeed, sponge settlers originating from heatwave-exposed sponges took longer to metamorphose than settlers originating from control sponges (figure 5). Larvae exposed to high temperatures within their physiological thermal threshold are known to accelerate their metamorphosis rate in several marine invertebrates [89–91], including sponges [92]. Delayed metamorphosis at high temperatures can be caused by a transient state of dormancy to survive the period of environmental stress [93]. Delayed osculum formation, and consequently the ability of juveniles to filter-feed, could be interpreted as an adaptive strategy by which *C. incrustans* can overcome extreme thermal events.

## 5. Conclusion

The quantity and quality of parental resources allocated to propagules can profoundly affect offspring growth and survival [94], and heritable bacteria have been shown to influence host tolerance to thermal stress in several organisms [95,96]. Overall, our study showed that marine heatwave conditions triggered carryover effects on the larval microbiome and the early life-traits of *C. incrustans*. Our results suggest the occurrence of selective vertical transmission as a potential mechanism for sponge adaptation to new environmental conditions and highlight possible developmental functions of vertically transmitted bacteria during post-settlement metamorphic phases in *C. incrustans*.

## Data Availability

Data and R scripts used in this manuscript are available on Dryad (https://doi.org/10.5061/dryad.f4qrfj70r) [97]. Electronic supplementary material are available on Figshare [98].
